# Methylmercury exposure in a subsistence fishing community in Lake Chapala, Mexico: an ecological approach

**DOI:** 10.1186/1476-069X-9-1

**Published:** 2010-01-11

**Authors:** Leonardo Trasande, Juanita E Cortes, Philip J Landrigan, Mary I Abercrombie, Richard F Bopp, Enrique Cifuentes

**Affiliations:** 1 Department of Community and Preventive Medicine, Mount Sinai School of Medicine, 1 Gustave L Levy Place, Box 1057, New York, NY 10029, USA; 2 Department of Pediatrics, Mount Sinai School of Medicine, 1 Gustave L Levy Place, Box 1057, New York, NY 10029, USA; 3 Instituto Mexicano de Tecnologia de Agua (Mexican Institute of Water Technology, IMTA), Paseo Cuauhnáhuac 8532, Colonia Progreso, CP 62550, Jiutepec, Morelos, México; 4 Environmental Health Unit, Instituto Nacional de Salud Publica (National Institute of Public Health, INSP), Universidad No 655, Col Santa María Ahuacatitlán, Cerrada Los Pinos y Caminera, Cuernavaca, Morelos CP 62100, México; 5 Department of Earth and Environmental Sciences, Rensselaer Polytechnic Institute, Jonsson-Rowland Science Center, 1W19, 110 8th Street, Troy, NY 12180, USA

## Abstract

**Background:**

Elevated concentrations of mercury have been documented in fish in Lake Chapala in central Mexico, an area that is home to a large subsistence fishing community. However, neither the extent of human mercury exposure nor its sources and routes have been elucidated.

**Methods:**

Total mercury concentrations were measured in samples of fish from Lake Chapala; in sections of sediment cores from the delta of Rio Lerma, the major tributary to the lake; and in a series of suspended-particle samples collected at sites from the mouth of the Lerma to mid-Lake. A cross-sectional survey of 92 women ranging in age from 18-45 years was conducted in three communities along the Lake to investigate the relationship between fish consumption and hair mercury concentrations among women of child-bearing age.

**Results:**

Highest concentrations of mercury in fish samples were found in carp (mean 0.87 ppm). Sediment data suggest a pattern of moderate ongoing contamination. Analyses of particles filtered from the water column showed highest concentrations of mercury near the mouth of the Lerma. In the human study, 27.2% of women had >1 ppm hair mercury. On multivariable analysis, carp consumption and consumption of fish purchased or captured from Lake Chapala were both associated with significantly higher mean hair mercury concentrations.

**Conclusions:**

Our preliminary data indicate that, despite a moderate level of contamination in recent sediments and suspended particulate matter, carp in Lake Chapala contain mercury concentrations of concern for local fish consumers. Consumption of carp appears to contribute significantly to body burden in this population. Further studies of the consequences of prenatal exposure for child neurodevelopment are being initiated.

## Introduction

Mercury is an ubiquitous environmental toxin. It exists in three general forms with different bioavailability and toxicity profiles -- the metallic element, inorganic mercury and organic mercury [1]. While coal-fired power plants and chloralkali plants are the leading point sources of mercury emissions in many industrialized countries [2,3], releases associated with amalgamation of precious metals may dominate in regions practicing artisanal mining of gold and silver [4]. Emissions from volcanoes are an important natural source and forest fires, often associated with clearing land for agriculture, can be significant [5,6] with disproportionate impacts in areas of current and recent deforestation [7-9].

Mercury is released from combustion sources in both elemental and inorganic forms. In the atmosphere, elemental mercury is converted to inorganic ("reactive") forms that eventually deposit into soil and water. Once in natural water systems, via direct deposition or terrestrial runoff, a portion of the mercury can be transformed to an organic form, methylmercury. The microbially-mediated process occurs both in sediments and in the water column [10]. Methylmercury is a potent neurotoxicant, especially to the developing brain [11,12]. It biomagnifies in aquatic food chains where "nearly 100% of the mercury that bioaccumulates in upper-trophic-level fish (predator) tissue is methylmercury" [13]. Highest concentrations are generally found in predatory fish at the top of the food chain - swordfish, tuna, king mackerel and shark in marine systems [14-17]; and black bass, walleye, and northern pike in freshwater systems [18]. Consumption of contaminated fish is the most important route of human exposure to methylmercury [19]. Studies in New Zealand, [20,21] the Faroe Islands, [11,22] and the Seychelles Islands [23] have followed cohorts to assess the impact of fetal methylmercury exposure. In a review of these three studies, the National Academy of Sciences (NAS) found strong evidence for neurotoxicity, even at relatively low exposure [24]. Since the NAS report, an American cohort has associated elevated hair mercury concentrations with decreases in cognition among infants. The association persisted even when controlled for maternal fish consumption [12].

**Figure 1:**
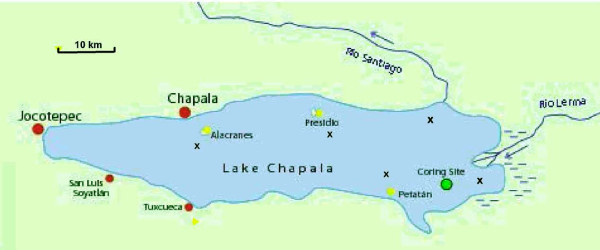
**Map of Lake Chapala and Study Locations**. Suspended particle sampling sites are shown as x's and named (Table 4) for nearby rivers (Lerma and Santiago) or islands.

Lake Chapala is the largest watershed in Mexico (Figure 1; Table S-3 in Additional file 1) and it collects water for one-eighth of all the irrigated land in Mexico. Some 300,000 people live in communities around Lake Chapala, and to varying degrees rely upon fish caught or purchased from the lake for their subsistence. Economic activities have created increasing pressure on the whole ecosystem prompting representatives from the concerned fishing communities to solicit an assessment of the health risk posed by industrial sewage discharges and agricultural practices (e.g., slash and burn). Previous reports of fish mercury concentrations (0.05-1.84 μg/g wet weight), some exceeding international guidelines (0.5-1.0 ppm), have raised serious concerns about health risks to families who rely on fish from the lake for their subsistence [25,26].

In response to increasing community concerns about potential health risks, an environmental sampling approach was used to study pathways of methylmercury exposure in this subsistence fishing population. Mercury contamination was assessed in carp, whitefish and tilapia from Lake Chapala collected in March 2007, and contamination with polychlorinated biphenyls and persistent pesticides was explored through analysis of the fish with the highest mercury contamination. Two sediment cores and eleven suspended-particle samples were collected from the lake and analyzed for total mercury to assess the importance of historical and current inputs.

To quantify human exposure, we conducted a cross-sectional survey of 92 women aged 18-45 years old in three communities that lie on the north, west and south shores of Lake Chapala (Chapala, Jocotepec, and Tuxcueca, respectively, Figure 1) in 2008, assessing fish consumption and its correlation with mercury concentration in hair samples from women of child-bearing age.

## Methods

### Environmental Specimen Collection

Samples of fish from Lake Chapala, including commonly consumed carp, charales (whitefish), and tilapia were obtained by purchase from local fishermen who were observed catching the fish from the lake. The samples were originally stored at 0-4°C, but warmed to ambient temperature during the two-day transport to Rensselaer Polytechnic Institute (RPI). Subsequent testing (see Table S-2, Additional file 1) indicated that such handling does not significantly affect total mercury concentrations. Samples of skinless filet from tilapia and carp as well as headless, eviscerated charales, typical representations of "edible portions" of the respective species, were analyzed for total mercury using direct mercury analysis on a Milestone DMA-80 following USEPA Method 7473 (sediments, soils, and sludges). The applicability of this method to analysis of fish flesh has been previously demonstrated [27]. Additional information specific to the analyses carried out in this study is presented in Additional file 1 (Figure S-3).

Sediment was sampled in two 28-30 cm push cores from the shallow marshy area at the east end of the lake, near the mouth of the Rio Lerma (Figure 1). Each core was divided into 2 cm sections, and maintained at room temperature throughout transport. Upon arrival at RPI, core sections were analyzed by gamma spectroscopy to identify "recent" deposition (i.e., within the last several decades). Selected core sections were also analyzed for total mercury to determine the general level of recent sediment contamination [28].

To assess "current" (i.e. 2007) concentrations of total mercury on particles in Lake Chapala, eleven samples of suspended material were obtained from four different locations on the lake (Figure 1) by filtering 250 to 780 mls of water through pre-fired quartz (Whatman QMA) filters. The samples were analyzed for total mercury by direct mercury analysis at RPI. This method was developed in the laboratory of one of the authors (R.B.) and is described in the Supplementary Material.

### Human Subjects and Setting

A convenience sample of 92 women were recruited as they came to three different public health clinics in communities located on the shore of the lake (Chapala, Tuxcueca and Jocotepec) for routine health care maintenance. Participation rate was not recorded, but fewer than five potential participants declined. All participants were fully informed about the purposes and limitations of the study and provided a written consent in Spanish. The study protocol was approved by the Research Review Board and the Ethics Committee from the Instituto Nacional de Salud Publica (National Institute of Public Health, INSP), and the Mount Sinai School of Medicine provided L.T. exemption from review for purposes of analyzing the already collected and deidentified data.

Food frequency questionnaires that have been validated in the Mexican population [29,30] were used to assess fish consumption and other components of diet in this population. In refining the questionnaire for the Lake Chapala population, the authors identified types of fish commonly consumed in the area (tilapia, charales, carp and catfish) as well as other fish and fish soup (sopa de pescado), and modified the questionnaire to determine the frequency with which the participants consumed each type of fish, and the place from which they obtained the fish (directly from the lake, purchase from a vendor who fishes from the lake, or elsewhere). Other parts of the questionnaire obtained demographic information as well as age, educational level, occupation, dental amalgam use, and home type.

### Hair Collection

Research assistants collected a sample of approximately 50-100 strands of hair from the occipital scalp of each woman, following a well-established sample collection procedure [31]. Samples were then enclosed in individual 5 × 10 cm resealable polyethylene bags, marked with identification numbers, and maintained at room temperature while transported to RPI for analysis.

Upon receipt at RPI, each hair sample was measured and rinsed with distilled deionized water. Stainless steel scissors were used to cut 2 cm from the proximal end of each sample; these 2 cm segments were placed in glass scintillation vials which had been prefired at 450°C and filled with approximately 10 mL of 1% Triton X-100 solution. Following Oken et al., (2005) the vials were sonicated for 15 min., then samples were rinsed several times with distilled deionized water and dried overnight. Vials were sealed with Teflon-lined caps and held at room temperature until analysis was performed.

In preparation for mercury analysis, caps were removed and vials were covered with a Kimwipe and kept inside a plexiglas glove box for several hours while relative humidity was maintained at 55 ± 5%. Stainless steel scissors were used to further cut 2 cm hair segments into pieces measuring 1-2 mm before transferring into quartz boats. Narrow strips cut from pre-fired quartz filter material (Whatman QMA) were pressed onto surface of samples to prevent loss during processing. Sample weights generally ranged from 10 to 20 mg, with the goal of conducting at least two mercury analyses on the first two cm of each woman's hair.

### Mercury Analysis

Analysis of total mercury content was performed by cold vapor atomic absorption spectrometry (CV-AAS) on a Direct Mercury Analyzer (DMA-80). Quality assurance was maintained by inserting a blank (an empty quartz boat) at the beginning of each sample run. Readings for blanks averaged an absorbance of .001, equivalent to ~0.04 ng of mercury. Blank readings were not significantly different from blank/blank (no sample/no boat) analyses which were run after each sample to certify that mercury carryover was negligible.

Aqueous standards and certified reference materials were included in each run, which typically contained nine samples. Certified reference materials utilized were IAEA-086 (International Atomic Energy Agency, Vienna, Austria) -- cryogenically homogenized human hair with total mercury of 0.573 ± 0.039 ppm, NIES CRM-13 (National Institute for Environmental Studies, Tsukuba, Japan) -- homogeneous human hair powder certified for total mercury of 4.42 ± 0.20 ppm, soil and sediment Standard Reference Materials and aqueous standards traceable to Certified Reference Solutions. The minimum quantification limit of the procedure is ~0.2 ng of total mercury, corresponding to ~20 ppb in a typical 10 mg hair sample. Additional information related to quality assurance of the hair analyses for total mercury can be found in Additional file 1 (Table S-3).

### Analysis for Persistent Organic Pollutants

The fish (a carp) with the highest mercury content was also analyzed for polychlorinated biphenyls (PCBs) and persistent pesticides as a probe for possible contamination with these ubiquitous pollutants within the ecosystem (Supplementary Material Section B). Analyses were performed by Axys Analytical Services, Ltd. using gas chromatography/low resolution mass spectrometry method MLA-028 (PCBs as Aroclors and Chlorinated Hydrocarbon Pesticides), developed by Axys in consultation with regulatory agencies including the USEPA and NYSDEC.

### Statistical Analysis

Descriptive, bivariate and multivariate analysis of hair mercury were performed, using Stata 10.0 (Stata Corporation, College Station, TX). Hair mercury concentration at the 0-2 cm length from the base was analyzed as the dependent variable. This represents approximately two months of hair growth and a similar timescale of dietary mercury exposure (see Figure S-2, Additional file 1). Fish consumption was collected as an ordinal variable (<1 time per month, 1-3 times per month, once per week, 2-4 times per week, 5-6 times per week or daily), but, upon examining the distribution of responses, was collapsed for purposes of bivariate and multivariable analysis to <1 meal per month, at least once per month but <1 meal per week, and ≥ 1 meal per week for tilapia, whitefish, other fish and fish soup. For carp and catfish, consumption was collapsed into <1 meal per month and ≥ 1 meal per month. Source of fish was analyzed as a categorical variable (purchase from a fisherman on or near Lake Chapala or capture of fish from Lake Chapala versus purchase from a supermarket or other vendor). Amalgam number was collapsed into none, 1-2 or >2.

**Figure 2:**
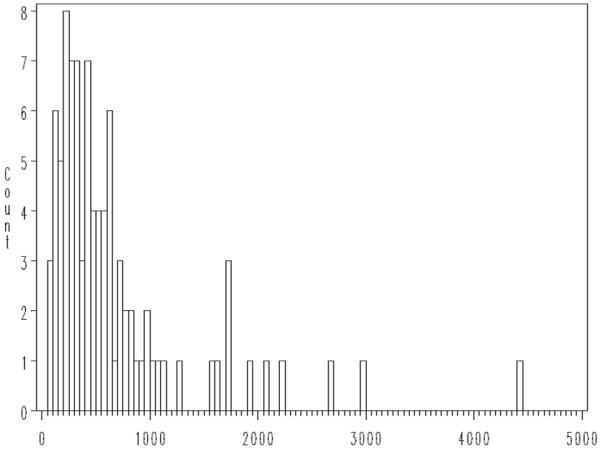
**Histogram of Hair Mercury Levels in Population Studied (Outlier of 141 ppm Excepted)**.

The distribution of hair mercury concentrations was found to be positively skewed (see Figure 2), with one outlier (141 ppm, confirmed on multiple analyses). This outlier was not included in bivariate and multivariate analysis to identify predictors of hair mercury concentrations. It should be noted, however that a similar "outlier," a mother with 86 ppm total hair mercury, was found in a New Zealand cohort [32]. The NHANES study reported three exceptionally high hair mercury concentrations, all in Mexican-Americans, 849 ppm in a woman, 415.2 ppm in a one-year old child, and 109.8 ppm in a three-year old [31].

Log transformation of the remaining data resulted in a distinctly normal distribution by Kolmogorov-Smirnov test. Bivariate analysis was performed for each of the above predictor variables as well as age and educational level using generalized linear modelling with a logarithmic link to account for the skewed distribution of hair mercury. Multivariable analysis was performed using each of the fish consumption variables and purchase or capture of fish from Lake Chapala as dependent variables. Amalgam number was not included in the multivariable analysis, as it was not significantly associated with hair mercury concentrations on bivariate analysis.

## Results

Table 1 presents mercury concentrations in the fish we analyzed. Among the species analyzed, carp not only have the highest concentrations of mercury (mean 0.87 ppm), but also the highest variability. The magnitude and variability of contamination of tilapia (mean 0.04 ppm) and whitefish (mean 0.11 ppm) were much less marked. A sample of the flesh from the carp with the highest mercury content (LLC 2, Table 2) was analyzed for PCBs and persistent pesticides. Total PCBs were 16 ppb (SDL <1 ppb), more than two orders of magnitude lower than the FDA tolerance level (2 ppm) and well within the range of "unlimited consumption, no advisory" category (<50 ppb) of the PCB advisory for the Great Lakes [33]. The most abundant chlorinated hydrocarbons, by far, were DDT-derived compounds. The total DDT level was 66 ppb (SDL <0.1 ppb), almost two orders of magnitude below the FDA tolerance level of 5 ppm. Furthermore, it is well below the screening value applied by the California Office of Environmental Health Hazard Assessment (100 ppb) indicating that consumption of fish contaminated at this level was judged unlikely to cause adverse health effects in humans [34].

**Table 1:** Total mercury levels (ppm, wet weight) in fish from Lake Chapala collected in March 2007.

Species	Number of samples	Mean	Median	Minimum	Maximum
Whitefish	13	0.11	0.10	0.07	0.15

Tilapia	14	0.04	0.04	0.03	0.06

Carp	6	0.87	0.50	0.25	2.44

**Table 2:** Data on individual carp from Lake Chapala.

Fish ID	Source^1^	Sex	Length^2 ^(mm)	Total Hg^3 ^(ppm wet)
LCC 1	Lake	Male	240	1.13 ± 0.13

LCC 2	Lake	Female	247	2.44 ± 0.43

LCC 3	Lake	Female	210	0.25 ± 0.03

LCC4	Lake	Not Reported	210	0.51 ± 0.04

LCC M1	Market	Female	180	0.39 ± 0.04

LCC M2	Market	Not Reported	265	0.50 ± 0.11

^1^Some carp were collected directly from the lake and others were purchased at a nearby market.

^2^Length of the filet provided; approximately 60% of the total length of the fish.

^3^Values are reported with one standard deviation based on multiple analyses.

Three samples from the upper twenty cm of sediment core Chap 2 had total mercury concentrations between 0.2 and 0.4 ppm dry weight (Table 3). In the 28-30 cm sample, the level was significantly lower, 0.07 ppm, a level within the range of background values reported for sediment cores [28]. Concentrations in core Chap 1, collected in the same general area (Figure 1), about 300 meters from Chap 2, were somewhat higher ranging from about 0.6 ppm in the upper 10 cm to a maximum of 1.3 ppm in the 18-20 cm section. The bottom section of the core, 24-26 cm, was still significantly above background at 0.6 ppm. Total mercury concentrations on "very recent" particles - the samples of suspended matter filtered from Lake water - ranged from 0.3 to 0.8 ppm (Table 4). The lowest concentrations of total mercury were seen in samples from the western most site (Alacranes, Table 4, Figure 1).

**Table 3:** Total mercury levels in sediment cores from the eastern end of Lake Chapala.

Core ID	Depth (cm)	Total Hg (ppm)	SD*
Chap 1	0-2	0.65	0.05

	2-4	0.69	0.05

	4-6	0.71	0.05

	6-8	0.78	0.04

	8-10	0.73	0.05

	10-12	0.82	0.06

	12-14	0.78	0.06

	14-16	0.86	0.07

	16-18	1.06	0.08

	18-20	1.28	0.10

	20-22	0.98	0.07

	22-24	1.18	0.11

	24-26	0.60	0.05

			

Chap 2	0-2	0.29	0.02

	8-10	0.20	0.02

	18-20	0.41	0.03

	28-30	0.07	0.01

* The relative SD based on analyses of 6-9 separate aliquots of 6 of these samples ranged from 5.7-9.7%. For the other twelve samples, the mean relative SD (7.5%) was assumed.

**Table 4:** Total mercury levels on suspended particles filtered from Lake Chapala in March 2007.

Site ID	Filter ID	Volume Filtered (ml)	Particle mass (g)	Total Mercury* (ng/g)
Santiago	DX 1	780	0.0272	480

Santiago	DX 2	460	0.0164	420

Lerma	DX 5	NR^#^	0.1174	670

Lerma	DX 6	250	0.0568	600

Alacranes	DY 5	530	0.0138	290

Alacranes	DY 6	225	0.0057	400

Presidio	DY 7	420	0.0101	770

Presidio	DY 8	610	0.0138	690

Petatan	DY 11	695	0.0434	580

Petatan	DY 12	340	0.0272	580

* Each filter was cut into two pieces that were analyzed separately for total mercury content. The value reported is the mean of the two measurements. In all cases except one, the relative deviation from the mean was <10%. For filter DY 7 (Presidio) it was 15%.

# NR = Not Recorded.

Results of hair mercury analysis are provided in Table 5. The mean hair mercury with the exception of the outlier was 686.4 ppb, and 27.2% of participants had hair mercury concentrations >1 ppm. On bivariate analysis, higher carp and whitefish consumption were strongly associated with increases in hair mercury concentrations. Consumption of fish purchased from a Lake Chapala fisherman or directly captured from the Lake showed a tendency towards elevated hair mercury concentrations (p = .077). Age and educational level were not associated with significant differences in mean hair mercury (p = .227 and .705 respectively, data not shown), as was residency in Chapala, Jocotepec or Tuxcueca. On multivariable analysis (Table 6), consumption of fish purchased from a Lake Chapala fisherman or directly captured from the Lake was associated with 399.1 ppb higher mean hair mercury concentration (95% CI: 107.0, 856.4), while ≥ 1 meal per month carp consumption was associated with a 436.1 ppb increase (95% CI: 75.1, 1063.4). No other fish consumption variable was associated with significant differences in hair mercury concentrations.

**Table 5:** Hair Mercury and Fish Consumption in Women of Childbearing Age in Three Lake Chapala Communities

Population	Mean (SD) and Median Hair Mercury Concentration ppb	Percent > 1 ppm
All participants (n = 92)	2218.0 (14707.5), 463.5	27.2%

Excepting outlier (n = 91)	686.4 (720.2), 456.7	26.4%

Fish consumption, amalgam use or demographic characteristics	Mean Hair Mercury Concentration (SD), ppb	Percent > 1 ppm

		

Resident of Chapala (n = 24)	727.7 (588.6)	29.2%

Resident of Tuxcueca (n = 40)	740.0 (874.9)	20.0%

Resident of Jocotepec (n = 29)	587.1 (612.9)	13.8%

		

Purchase fish from fisherman on Lake Chapala or catch fish from Lake Chapala (n = 34)	856.0 (914.4)	23.5%

Purchase fish at supermarket, street vendor or other location (n = 57)	585.3 (559.6)	12.3%

		

No dental amalgams (n = 55)	732.2 (795.2)	20.0%

1-2 dental amalgams (n = 14)	735.7 (781.3)	21.4%

>2 dental amalgams (n = 22)	540.8 (435.5)	4.5%

		

<1 time/month carp consumption (n = 47)	526.0 (536.2)	10.6%

≥ 1 time/month carp consumption (n = 44)	857.8 (848.5)*	22.7%

		

<1 time/month whitefish consumption (n = 49)	550.9 (474.7)	14.3%

≥ 1 time/month but <1 time/week whitefish consumption (n = 30)	825.7 (897.8)	20.0%

≥ 1 time/week whitefish consumption (n = 8)	1085.7 (1148.6)*	25.0%

		

<1 time/month fish soup consumption (n = 32)	603.8 (467.8)	15.6%

≥ 1 time/month but <1 time/week fish soup consumption (n = 33)	685.0 (619.5)	18.2%

≥ 1 time/week fish soup consumption (n = 22)	897.7 (1100.7)	18.2%

		

<1 time/month tilapia consumption (n = 15)	703.7 (590.0)	26.7%

≥ 1 time/month but <1 time/week tilapia consumption (n = 33)	455.4 (452.2)	6.1%

≥ 1 time/week tilapia consumption (n = 39)	863.0 (884.7)	20.5%

		

<1 time/month catfish consumption (n = 52)	576.7 (656.3)	13.5%

≥ 1 time/month catfish consumption (n = 31)	868.5 (821.0)	22.6%

		

<1 time/month other fish consumption (n = 47)	683.5 (772.9)	14.5%

≥ 1 time/month other fish consumption (n = 44)	695.8 (537.1)	22.7%

* p < .05, ** p < .01, *** p < .0001

**Table 6:** Multivariable Analysis of Hair Mercury Concentration in Women of Childbearing Age in Three Lake Chapala Communities

Fish consumption, amalgam use or demographic characteristics	Increment in Mean Hair Mercury Concentration (95% CI), ppb
Purchase fish from fisherman on Lake Chapala or catch fish from Lake Chapala	399.1 (107.0, 856.4)**

Purchase fish at supermarket, street vendor or other location	Reference

	

<1 time/month carp consumption	Reference

≥ 1 time/month carp consumption	436.1 (75.1, 1063.4)**

	

<1 time/month whitefish consumption	Reference

≥ 1 time/month but <1 time/week whitefish consumption	119.1 (-100.0, 493.5)

≥ 1 time/week whitefish consumption	-155.1 (-278.8, 86.3)

	

<1 time/month fish soup consumption	Reference

≥ 1 time/month but <1 time/week fish soup consumption	-74.8 (-224.9, 197.4)

≥ 1 time/week fish soup consumption	136.6 (-69.4, 467.6)

	

<1 time/month tilapia consumption	Reference

≥ 1 time/month but <1 time/week tilapia consumption	-224.8 (-329.0, 14.8)

≥ 1 time/week tilapia consumption	-155.1 (-278.8, 86.3)

	

<1 time/month catfish consumption	Reference

≥ 1 time/month catfish consumption	73.6 (-147.4, 481.2)

	

<1 time/month other fish consumption	Reference

≥ 1 time/month other fish consumption	-44.6 (-167.2, 441.6)

* p < .05, ** p < .01, *** p < .0001

## Discussion

Our major findings from this pilot study of subsistence fishing population in Lake Chapala, Mexico are that (1) contamination of fish, especially carp, is significant, and (2) consumption of contaminated fish, especially carp, contributes significantly to hair mercury concentrations in women of childbearing age, a population of significant public health concern because of the potent prenatal neurotoxicity of methylmercury.

Concentrations of mercury in the small population of Lake Chapala carp we examined were surprisingly high, even in comparison to the upper Hudson River, a system known for high concentrations of mercury contamination [28]. Total mercury concentrations in analogous samples (i.e. skinned filets) from upper Hudson carp of similar to significantly larger size ranged from 0.08 to 0.58 ppm (n = 78; median = 0.26 ppm) [18]. It is likely that the high concentrations of mercury found in Lake Chapala carp (Table 2) are related to their particular feeding habits in the lake. It has been reported that the species of carp introduced to Lake Chapala over a hundred years ago fed extensively on whitefish eggs and fingerlings [35]. While carp are generally omnivorous, feeding significantly on plant and animal material extracted from bottom sediment, enhanced piscivorous behavior in Lake Chapala provides a most reasonable explanation for the highly elevated concentrations of total mercury relative to upper Hudson carp.

The upper 10 cm of sediment are generally considered the "biologically active" zone where physical and biological mixing and the feeding of benthic organisms introduce persistent contaminants to the foodchain. This is particularly true in shallow marshy areas where carp spawning has been found to effect complete mixing of the sediments to approximately this depth [28]. Our sediment cores were collected from such an area at the eastern end of Lake Chapala (Figure 1). Concentrations in the upper 10 cm of the cores and also in the suspended particle samples ranged from about 0.2 to 0.6 ppm, suggesting that ongoing contamination of Lake Chapala is moderate. Thirteen published studies [36] report background concentrations of total mercury in freshwater sediments that range from 0.01 to 0.21 ppm. For additional perspective, recent sediments from the NY/NJ Harbor deposited between the 1960s and mid 1990s had total mercury concentrations ranging from about 1 ppm to more than 10 ppm while less industrialized areas of the Hudson basin typically contain a few tenths of a ppm total mercury in recent deposition [28]. A study of atmospheric deposition of mercury in seven lakes located within the forest preserve of the Adirondack Mountains in NYS found concentrations between 0.2 and 0.6 ppm in recent sediments [36]. In our Lake Chapala cores, the level of total mercury reached approximately 1 ppm at depths between 16 and 24 cm in core Chap 1 (Table 3). This suggests that historical fluxes of mercury to the lake were significantly greater than current fluxes. Low activities of excess Pb-210 and Cs-137, the radionuclides most commonly employed in chronological studies of recent lake sediments, and the likelihood of a complex sedimentological history at the site related to fluctuating lake concentrations, prevented any meaningful radiodating of these cores. The short lived natural radionuclide, Be-7, was detected in the surface (0-2 cm) section of Chap 2 indicating that the site had received at least some very recent deposition, more specifically that a significant fraction of the particles in the upper two centimeters were deposited within about a year of core collection [28]. It is planned to develop a several decades long chronology of mercury concentrations in Lake Chapala from sediment cores that will be collected from the deepest part of the lake, a sedimentary environment expected to have more consistent and continuous deposition with limited biological and physical mixing, conditions more amenable to radiodating [28].

The average level of total mercury on particles filtered from Lake Chapala water collected at our western most site (345 ng/g at Alacranes, Figure 1) was more than two standard deviations lower than the mean at the other four sites (599 ± 117). This suggests further investigation of a possible source of contamination to the eastern end of Lake Chapala, perhaps associated with inputs from Rio Lerma, the largest tributary. Our technique of measuring total mercury on particles filtered from a relatively small volume of water is ideally suited to rivers such as Rio Lerma where sampling along a transect can be used to identify and evaluate point sources. In addition, there are several primary sources of mercury in the Lake Chapala basin that warrant further investigation. These include local and regional volcanism, the mining of cinnabar (HgS) in Michoacan, the Mexican state bordering the southern end of Lake Chapala, and historical artisanal gold mining in the drainage basin [37].

Our analysis of hair mercury concentrations in Lake Chapala suggests significant bioaccumulation in women of child bearing age in this population and raises the potential for neurodevelopmental impact on their offspring. While hair mercury concentrations we detected were on average lower than those detected in the Faroe Islands (median 4.3 ppm) [11] and Seychelles (median 6.3 ppm) cohorts, [23] Lake Chapala women appear to have higher hair mercury concentrations than a US reference population (median 0.2 ppm) [31]. Our review identified only one other study of a Mexican population that assessed hair mercury. In a sample of 47 women and children from Veracruz near the Gulf of Mexico, 58% were found to have hair mercury concentrations above 1 ppm [38]. Other studies of fish and sediment mercury concentrations in Mexico have largely been performed in coastal areas near the U.S. border, [38-43] where mechanisms of contamination appear to differ from that experienced in Lake Chapala. It will also be important to assess other routes of exposure to mercury, as studies have documented the use of mercury-contaminated dietary supplements [44] as well as mercury-containing personal care products[45] and medicinal compounds [46].

The data we collected suggests that the population examined is not purely a subsistence fishing population in the sense that fish are used to maintain or support the whole population at a minimum level. We recognize that other aspects of diet (especially fruit) have also been documented to influence mercury levels in fish consuming populations [47,48]. More careful comparison to US NHANES data suggests that hair concentrations in our population were similar to women who were frequent fish consumers (>3 fish in the past 30 days) of similar age (median 340 ppb, mean 770 ppb) [31]. We collected but chose not to examine other aspects of diet in this pilot study due to the limited sample size, but ongoing studies will assess different components of diet more carefully to examine the degree to which participants are relying on fish from Lake Chapala for subsistence and to assess the role of other aspects of diet in influencing bioavailability of orally consumed methylmercury. It is also possible that consumption of large amounts of smaller fish (e.g., whitefish) in a given meal may not result in a meaningful difference in mercury exposure than that which would result from consumption of one large carp. Before proceeding to intervene and shift consumption in the population from carp to other Lake Chapala fish, ongoing studies are quantifying fish consumption to account for number of fish in a given meal and weight of fish consumed.

Further studies are needed to examine whether elevated hair mercury concentrations in the range experienced in the Lake Chapala population reflect significant risks for exposure to the developing brain, and to confirm the absence of potential confounding neurotoxicant exposures such as lead, arsenic and pesticides. The absence of "concentrations of concern" of PCBs in the fish with the highest mercury concentration lowers the likelihood of risks posed by this group of contaminants. Testing of human serum could rule out this confounder definitively. The usual caveats about generalizing findings from small samples are important, and these findings merit further confirmation in a larger cohort of women of childbearing age and through collection of sediment cores from the center of the lake as well as collection of additional suspended particle samples at various locations along the Rio Lerma to locate potential point sources of mercury. While a cohort study of children born to exposed women would confirm whether this level of methylmercury exposure is problematic for neurodevelopment in this population, proactive public health guidance about the potential risk posed by consumption of carp among vulnerable populations seems warranted.

## Conclusions

Despite a moderate level of contamination in recent sediments and suspended particulate matter, carp in Lake Chapala contain sufficient mercury concentrations to suggest that it contributes significantly to body burden in this population. While ongoing research is needed, our results suggest concern for prenatal methylmercury exposure and its implications for child neurodevelopment in this population.

## List of Abbreviations

CV-AAS: Cold Vapor Atomic Absorption Spectrometry; DMA: Direct Mercury Analyzer; IAEA: International Atomic Energy Agency; INSP: Instituto Nacional de Salud Publica; NAS: National Academy of Sciences; NIES: National Institute for Environmental Studies; NYS: New York State; PCB: Polychlorinated Biphenyl; RPI: Rensselaer Polytechnic Institute.

## Competing interests

The authors declare that they have no competing interests.

## Authors' contributions

LT and EC conceived the study and obtained primary funding. LT and JC collected the primary data. Analyses of hair and environmental samples were performed by MA and RFB. Statistical analysis was performed by LT, MA and RFB. LT, EC, PJL and RFB reviewed and refined the manuscript. All authors read and approved the final manuscript.

## Supplementary Material

Additional file 1 **Methodological Details of Mercury Analytic Methods**. This file describes additional details regarding analytic methods for hair, sediment and filtered water samples collected in the present study. Quality assurance methods are also explained. Click here for file
