# Supplementary material for: Methylmercury exposure in a subsistence fishing community in Lake Chapala, Mexico: an ecological approach
Source: Environ Health. 2010 Jan 11;9:1. doi: 10.1186/1476-069X-9-1 (PMC2820022; doi:10.1186/1476-069X-9-1)
Supplement: Additional file 1 — Methodological Details of Mercury Analytic Methods. This file describes additional details regarding analytic methods for hair, sediment and filtered water samples collected in the present study. Quality assurance methods are also explained. [file 1476-069X-9-1-S1.DOC]

**Supplementary Material**

Mary Abercrombie and Richard F. Bopp

**A) MERCURY MEASUREMENTS**

**Introduction**

Additional details related to DMA-80 measurements of total mercury on the wide range of media analyzed as part of this study are reported below.

**Standard Curves**

A typical set of standard analyses covering the range of total mercury quantification is shown as Figure S-1. Best-fit curves (second order polynomial) used to determine concentrations in hair, fish, and suspended matter samples were based on analyses of both the aqueous standards (dilutions of Spex Certi-Prep PLHG2-1Y, 100 mg/l mercury) and soil and sediment SRMs (NIST SRM 2702, 2704, and 2709).

**Hair SRMs**

Direct applicability of the standard curves to our analyses of total mercury levels in hair was demonstrated with multiple analyses of IAEA-086 (International Atomic Energy Agency, Vienna, Austria) — cryogenically homogenized human hair, and NIES CRM-13 (National Institute for Environmental Studies, Tsukuba, Japan) — homogeneous human hair powder. The results are reported in Table S-1 and indicate that the precision and accuracy of the analyses are on the order of 10%, comparable to the error reported with the certified hair standards.

Static electricity makes the processing of hair at relative humidity (RH) less than 40% extremely challenging. Consequently, we have adopted a procedure that includes equilibrating the hair samples in a plexiglas® glove box at 55 ± 5 % RH. Our experiments gave an average of 10.8 weight % water (range of 10.3 to 11.2%) in four analyses of NIES CRM-13 equilibrated at between 50 and 60% RH. This is in excellent agreement with reported values for human hair of between 10 to 12 weight % water over this range of RH (El-Shimi and Princen 1978).

The certified values for both hair SRM are given as % dry weight. Given the 5 to 7 % relative uncertainties in the certified values (Table S-1) and the fact that drying a separate subsample of each hair sample would require significantly more effort than the total mercury analysis, we have chosen to report values on the basis of weight equilibrated at 55 ± 5 % RH. Consequently, our concentrations of total mercury in hair samples are a bit conservative, approximately 10% lower than on a dry weight basis.

**Response Time of Hair Mercury Levels**

One of the authors (RFB), a minimal fish consumer, shaved his head numerous times to provide samples for total mercury analyses. After establishing a fairly typical and consistent “background” level of ~200 ppb, a mercury “spike” was administered in the form of two meals of tuna consumed on consecutive days. Peak mercury levels in his hair occurred within two months and declined with a half time on the order of a month (Figure S-2). More controlled experiments are underway.

**Analyses of Fish for Total Mercury**

Direct applicability of the standard curves to our analyses of total mercury levels in fish was demonstrated by an inter-laboratory comparison exercise with the NYSDEC. Archived frozen aliquots of twenty-nine samples of fish flesh that had been analyzed for total mercury using the acid digestion method based on EPA Method 245.6 (Determination of Mercury in Tissues by Cold Vapor Atomic Absorption Spectrometry, Revision 2.3 (April 1991)) were supplied by Dr. Ronald Sloan of NYSDEC and analyzed at RPI on the DMA-80. The excellent agreement that was obtained (Figure S-3) supports the conclusions of Haynes et al. (2006).

The fish samples analyzed in this study were inadvertently warmed to ambient temperature for approximately two days during shipment from Mexico to RPI. To determine if such handling influenced the total mercury measurements, samples of yellowfin tuna, seabass, and swordfish were obtained from markets in the vicinity of RPI. Multiple aliquots of each were analyzed fresh and after sitting out in the laboratory for two to three days. The results (Table S-2) indicate that there were no significant changes in the measured total mercury levels.

**Analyses of Suspended Particles for Total Mercury**

The DMA-80 is particularly well suited to the analysis of total mercury in suspended particulate matter samples obtained by filtering a few hundred milliliters of water from lakes, reservoirs, or rivers. The filter material that we employ is Whatman QMA®, quartz microfiber which captures the great majority of the mass of suspended material. This filter material, when pre-combusted at 450°C, our standard procedure, gives total mercury levels that are indistinguishable from blanks. Finally, being quartz (the same material as the sample boats), QMA filters do not melt or decompose during the analysis which employs temperatures up to 850°C in the sample furnace.

Our procedure employs standard 50 mm diameter filters which yield two separate total mercury analyses. A 25 mm punch of the filtered material (56% of the filter-cake area obtained on a standard 47-50 mm filtering apparatus), can be easily packed into a quartz sampling boat. A second analysis can be done on the “remainder” (46%). We have tested this procedure on a stream and reservoir in New Jersey and on the Hudson and Mohawk Rivers in New York State. In general, suspended matter collected on the filter is distributed quite homogeneously. Typically the two total mercury analyses from a single filter of a given sample agree to within 10%.

**Figure S-1.** Typical standard curves for total mercury analyses on the DMA-80 (a) from 0 to 45 ng and (b) 45 to 600 ng based on aqueous standards and soil and sediment SRMs.

**Figure S-2.** Total mercury levels in hair shaved from the head of one of the authors (RFB) before and after two meals of tuna.

**Figure S-3.** A comparison of total Hg measurements in fish analyzed by NYSDEC following EPA Method 245.6 and at RPI using direct mercury analysis (DMA-80). Error bars represent one standard deviation based on multiple analyses of four of the samples at RPI.

**B) MERCURY AND PCBs IN FISH FROM THE UPPER HUDSON RIVER**

The New York State Department of Environmental Conservation has compiled a most extensive database on levels of persistent contaminants, including total mercury and PCBs, in fish and other wildlife (NYSDEC, 2008). Enhanced bioconcentration of mercury in predatory fish at the top of a freshwater foodchain is illustrated by data collected in the 1990s from Griffin Island (Figure S-4), a major wildlife sampling site for the upper Hudson River PCB Reassessment under Federal Superfund.

Although average total mercury concentrations were significantly lower in carp than in the top predatory fish, average total PCB concentrations were higher. This same pattern also occurs in the fish data from Onondaga Lake (NYSDEC, 2008), another Superfund site with both mercury and PCB contamination. It is also noteworthy that while the correlation between total mercury and total PCBs in the carp from Griffin Island was poor (R^2^ = 0.24; n = 15), the carp with the two highest levels of total mercury had the fourth and third highest levels of total PCBs, respectively. These observations support our analysis of the carp sample with the highest total mercury level as a probe for PCB and chlorinated hydrocarbon contamination in the Lake Chapala system.

**Figure S-3.** Total mercury levels in samples of fish (skinned filets and whole fish minus the head and viscera) from the upper Hudson River at Griffin Island (NYSDEC, 2008). Data from higher predators (black bass (smallmouth, SM; and largemouth, LM) and walleye) are plotted on the right. Data plotted are average values with 1 σ error bars. A single sample from a Northern Pike had 0.52 ppm total mercury, consistent with the data from the other high predators.

**C) THE LAKE CHAPALA BASIN**

Statistical information on Lake Chapala and its basin is presented in Table S-3.

**References**

Aparicio J. 2001. "Hydrology of the Lerma-Chapala Watershed". In The Lerma-Chapala Watershed, ed. Anne M. Hansen and Manfred van Afferden, 3-30. New York: Kluwer Academic/Plenum.

de Anda J, Quinones-Cisneros SE, French RH, Guzman M. 1998. Hydrologic Balance of Lake Chapala (Mexico). J. Am. Water Resources Assoc. 34:1319-1331.

El-Shimi, A.F. and H.M. Princen,  1978.  Water vapor sorption and desorption behavior of some keratins. Colloid & Polymer Science 256, 105-114.

Haynes, S., et al., 2006. An Evaluation of a Reagentless Method for the Determination of Total Mercury in Aquatic Life. Water, Air, & Soil Pollution. 172**,** 359-374.

NYSDEC. 2008. Hudson Basin Biota Database on Contaminants. FoxPro Version 6. Bureau of Habitat, Division of Fish, Wildlife, and Marine Resources, New York State Department of Environmental Conservation, Albany, New York. Updated August, 2008.
